# Self-Supervised Joint Learning Fault Diagnosis Method Based on Three-Channel Vibration Images

**DOI:** 10.3390/s21144774

**Published:** 2021-07-13

**Authors:** Weiwei Zhang, Deji Chen, Yang Kong

**Affiliations:** Key Laboratory of Embedded System and Service Computing, Tongji University, Shanghai 201804, China; zhangww@tongji.edu.cn (W.Z.); yangkong2012@gmail.com (Y.K.)

**Keywords:** self-supervised learning, fault diagnosis, three-channel vibration images, bearing

## Abstract

The accuracy of bearing fault diagnosis is of great significance for the reliable operation of rotating machinery. In recent years, increasing attention has been paid to intelligent fault diagnosis techniques based on deep learning. However, most of these methods are based on supervised learning with a large amount of labeled data, which is a challenge for industrial applications. To reduce the dependence on labeled data, a self-supervised joint learning (SSJL) fault diagnosis method based on three-channel vibration images is proposed. The method combines self-supervised learning with supervised learning, makes full use of unlabeled data to learn fault features, and further improves the feature recognition rate by transforming the data into three-channel vibration images. The validity of the method was verified using two typical data sets from a motor bearing. Experimental results show that this method has higher diagnostic accuracy for small quantities of labeled data and is superior to the existing methods.

## 1. Introduction

Rotary machinery is widely used in helicopters, engines, turbines, and other mechanical equipment, and is a vital component in industrial applications. As one of the most common parts in rotating machinery, bearings are widely used in the industry. Modern industrial production environments are harsh and variable. As a result, bearings are often used in difficult working environments. Bearing defects, if not rapidly detected, can cause unnecessary mechanical shutdowns, economic loss, and risks to personal safety. Therefore, monitoring of bearing health is important for the safe and reliable operation of rotary mechanical equipment and production.

At present, commonly used fault diagnosis methods are based on analysis models, knowledge, and data-driven. However, it is difficult to establish an accurate mathematical model or a complete empirical knowledge base for the fault diagnosis of complex mechanical systems. Data-driven fault diagnosis, which does not rely on mathematical models or expert experience, is a better choice in industrial practice. Signal acquisition, feature extraction and selection, and fault classification are the three main steps of the data-driven fault diagnosis method. First, in the process of signal acquisition, it is usually necessary to measure vibration, temperature, sound wave, current, etc. Among these signals, vibration signals are most widely used as they are relatively easy to measure and provide the most basic information about mechanical failures. Second, the original signal features are extracted by signal processing techniques, such as those in [1,2,3,4], and artificial feature selection is carried out. Finally, machine learning methods, such as artificial neural network (ANN), support vector machine (SVM), and random forest are used to classify faults and perform a health diagnosis of the rotating machinery. Although these methods have achieved positive results, defects remain. First, the traditional intelligent diagnosis model based on shallow machine learning cannot automatically learn the characteristics of complex classification tasks. Therefore, additional feature engineering processing is an essential process, and requires a significant amount of labor input and engineering experience. Second, due to poor generalization ability, features selected with a strong specific relevance cannot be applied to situations outside a specific scenario. Third, the characteristics of artificial selection are often based on high dimension data, which leads to over-fitting of the model.

Intelligent fault diagnosis methods based on deep learning have become a popular research topic. Diagnosis methods based on deep learning can be used to effectively classify fault states from recorded data samples without manual intervention. However, deep learning-based fault diagnosis is achieved using a supervised learning framework, which not only requires a large amount of labeled data, but ignores the fact that most of samples do not include marker information. Supervised learning is only suitable in situations in which a large number of labeled samples can be obtained [5,6]. The lack of a large amount of data annotation for supervised training can often result in poor training results from these algorithms. In mechanical fault diagnosis, sample labeling is highly time-consuming and expensive, and it may be impossible to obtain a large number of samples. Therefore, fault diagnosis methods based on supervised learning are not commonly used in practice.

Compared with that of supervised learning, the accuracy of unsupervised learning in the absence of labeled data and any distinguishing information is usually lower. In addition, unsupervised learning is unable to perform classification or recognition tasks well due to the lack of distinguishing information. Moreover, in the field of fault diagnosis, the accuracy of diagnosis is essential for the maintenance and operation of machinery, and human safety. To address the problem of the low accuracy of unsupervised learning, researchers proposed semi-supervised learning. The semi-supervised method combines the unsupervised and supervised learning techniques to train labeled and unlabeled data in a model, thereby improving the accuracy of recognition. Semi-supervised learning simultaneously addresses the limitations of supervised and unsupervised learning, and is a feasible scheme for fault diagnosis. However, in previously reported semi-supervised fault diagnosis methods [7,8,9,10], unsupervised pre-training was carried out on the unlabeled dataset, and supervised fine-tuning was then carried out on the resulting learned features. For example, unsupervised sparse filtering for feature extraction and a softmax classifier for fault diagnosis, which is a two-stage intelling diagnosis method, was proposed by Lei et al. [11]. A fault diagnosis method for transmission and rolling bearings of an electric locomotive based on an automatic encoder, which uses a combination of the automatic encoder and softmax to identify the fault state, was developed by Shao et al. [12]. The key to the above methods is the separate handling of labeled and unlabeled data, which is not consistent with semi-supervised learning.

To address the problem of data annotation and improve the learning of unlabeled data, numerous self-supervised learning methods have been recently proposed and summarized [13,14,15,16]. The basis of self-supervised learning is the acquisition of pseudo-tags with unlabeled data via the setting of auxiliary tasks. These pseudo-tags do not artificial annotation and can be generated using the image or video attributes; these false tags are then used to learn the characteristics of untagged data [13,16]. Self-supervised learning can be divided into many forms according to the research goal of the auxiliary tasks [14], including generative grammar, contrastive grammar, and generative grammar. Researchers have also found that self-monitoring can not only learn the representation of an unlabeled dataset without the need for manual annotation monitoring, but can also significantly improve the accuracy of the model, also, even in a fully labeled dataset [15]. However, self-supervised learning is usually divided into two stages: the first stage is to learn the potential features of unlabeled data via auxiliary tasks, and the second stage is to transfer the features learned in the first stage to other downstream tasks, for example, image classification, target detection, and segmentation. This multi-stage self-supervised learning method suffers from two problems: (1) it increases the complexity of the model, and (2) the pre-training features of the unlabeled data cannot be fully applied to the downstream tasks. Therefore, identification of approaches to effectively use the potential characteristics of self-supervised learning to complete the downstream tasks has received attention among researchers [14,15]. Considering the above problems, this paper combines self-supervised learning with the fault diagnosis task, and proposes a fault diagnosis method that combines self-supervised learning with hyper-visual learning. This method uses self-supervised learning to extract general features of fault samples from unlabeled samples, and to extract detailed features of fault samples from labeled samples by supervised learning. In addition, a training method that combines the self-supervised loss with the supervision loss was adopted. Among these, the supervised cross-entropy loss operation for labeled samples can significantly improve the recognition performance. In addition, further improvement can be made in the self-supervised MSE reconstruction loss operation and counter-loss operation for the labeled distribution of original unlabeled samples, model recognition performance, and generalization ability. The health status of the machine was diagnosed according to the coding features of the marked vibration samples. Taking a motor bearing as an example, the effectiveness of the method was verified. Compared with other methods, the method presented in this paper achieved remarkable results. In conclusion, the main contributions of this paper are as follows.

(1) A new fault diagnosis method based on self-supervised joint learning and three-channel vibration images is proposed;
(2) To improve the existing multi-stage self-supervised learning approach, an end-to-end self-supervised learning method is proposed that simplifies the model training process;
(3) Automatic fault feature extraction and learning with self-supervised learning can not only avoid the process of artificial feature extraction in traditional fault diagnosis methods, but also improve the robustness of the model;
(4) A new method for constructing three-channel vibration images is proposed that has competitive performance compared with commonly used data processing methods;
(5) We combine self-supervised learning with fault diagnosis, and the obtained results are superior to those obtained with previous methods based on supervised learning and semi-supervised learning.

The remainder of the paper is organized as follows. In Section 2, the basic theories used in this paper are briefly introduced. Section 3 details the method presented in this paper. Section 4 introduces the performance evaluation of the proposed method and presents experimental verification. Finally, Section 5 provides the conclusion of this paper.

## 2. Related Theory

### 2.1. Time-Domain 2D Image Conversions

According to both theory and experience, traditional 1D analysis is not usually able to capture the inherent mode of fault conditions. In the learning process [17,18], it is more appropriate to use images to represent information. In addition to time-frequency analysis, 1D original fault signals can be directly converted into 2D gray images [19]. Chong [20] proposed a method to transform the 1D vibration signal into 2D gray images, which can be used to effectively extract the fault characteristics of rotating machinery.

**Figure 1:**
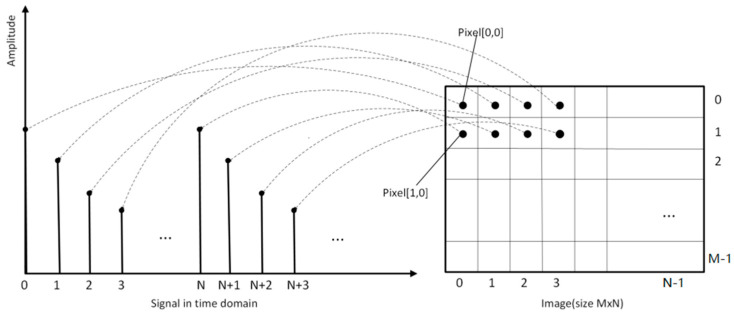
Vibration signal to image translation scheme.

Figure 1 provides a visual explanation of the transformation. In the graph, the number of samples contained in the vibration signal is M × N, where M × N represents the size of the image (M and N values are the rows and columns of the image, respectively). The values of M and N depend on the length of the vibration signal. The computational complexity of the method is directly proportional to these values. Therefore, M and N values should be selected to be as small as possible to reduce complexity; however, it should also be ensured that these values are sufficiently large to retain the most important features of the original. The values suggested by Chong [20] for M and N are: M = 128, 256, or 512, and N = 128, 256, or 512. The conversion process of the time-domain 2D image is clearly shown in Figure 1.

As shown in Figure 1, the coordinates of the corresponding pixels of sample i in the vibration signal are pixel (j,k), where j=floor (i/N), and K=modulo (i/N).

### 2.2. Self-Supervised Learning

To avoid time-consuming and expensive data annotation in supervised learning, the researchers propose a self-supervised learning method that can learn visual features directly from large-scale unlabeled data without any manual annotations. The pretext task is the basis of self-supervised learning. To date, various pretext tasks of self-supervised learning have been proposed, including gray image coloring [21], image repairing [22], jigsaw puzzle [23], and other methods [24,25,26].

Self-Supervised Learning Steps:

(1) In the self-supervised training stage, a pre-defined pretext task that needs to be solved is designed, and a pseudo-label for the pretext task is automatically generated according to some attributes of the data. Then, the model is trained to learn the objective function of the pretext task;
(2) Following the self-supervised training, the learned visual features can be used as a pre-trained model to be further transferred to downstream tasks to improve downstream task performance and overcome overfitting problems.

## 3. Proposed Methods

In this section, the SSJL fault diagnosis method based on three-channel vibration images is introduced in detail. First, the original vibration signal is transformed into a three-channel vibration image by the data processing module and, second, the fault feature is extracted by the self-supervised learning module of the SSJL method. Finally, the unlabeled samples and labeled samples are jointly studied and optimized by the joint learning module of the SSJL method.

### 3.1. Data Processing

**Figure 2:**
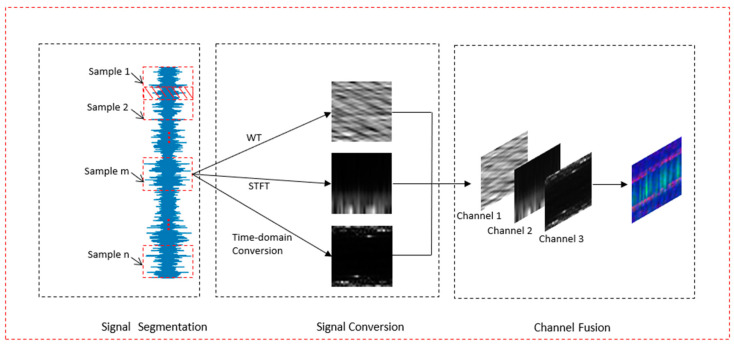
Three-channel RGB vibration image conversion process.

In the field of fault diagnosis, most algorithms use single time-frequency analysis technology to transform the two-dimensional gray image into a single-channel image as the input, or directly take the original vibration signal as the input. These methods have the following shortcomings: (1) the fault features of single-channel vibration images are not abundant compared with those of three-channel RGB images; (2) the time-frequency analysis technique describes the trend change in the spectrum content of the signal with time, which may lead to information loss and incompleteness of the original time-domain signal; and (3) previously, only a single time-frequency analysis technique was used—it is difficult for a single time-frequency analysis technique to encompass the characteristics of different faults. Therefore, this paper presents a new data processing method for constructing three-channel RGB vibration images. To make full use of the original time and frequency domain information, the vibration image is transformed into a three-channel RGB image, thus improving the feature distinguishability and information richness of the input data. The process of the data processing method includes three steps: signal segmentation, signal conversion, and channel fusion. Figure 2 shows the process of this method.

As can be seen from Figure 2, in the signal segmentation stage, we need to segment the original signals of different fault types according to a fixed length. The segmentation length of the signal is related to the period of the original signal, and it needs to meet three conditions: (1) the segment length should contain at least two cycles of the fault signal (fault signal periodically, with complete fault information included in each cycle); (2) the upper limit of the segmentation length needs to be adjusted appropriately according to the requirements of specific actual scenes and different data sets. The excessive value of this value will lead to excessive-resolution of the final three-channel color image, thus affecting the training and diagnosis time of the model. At the same time, it should also ensure that the number of samples after segmentation is sufficient; and (3) the number of samples contained in the segmentation length should be equal to the product m × n of the length and width of the two-dimensional image transformed by time-domain conversion, where m × n denotes the size of the image (m and n denote the rows and columns of the image, respectively). In this paper, to expand the training data set while also reducing the complexity, we set the segmentation length to 1024 and the segmentation process to overlap. Finally, we used resize to scale the image.

It is worth noting that the resulting images are slightly different due to different segmentation lengths. However, no matter how it changes, as long as the above three conditions are met, the final three-channel color image preserves the information of the overall fault characteristics; at the same time, the difference in the same fault signal is much smaller than the difference between the different faults themselves; for supervised learning, the difference is classified as the same type of fault by the label that is marked in advance, thus prompting the model to learn the difference as the same type of fault. In the case of self-supervised learning, the learning itself detects potential universal fault feature. This variability also increases the diversity of samples; the model based on a deep convolution network has translation invariance, so it can recognize the object correctly even if its appearance has changed due to shifting. Therefore, the difference in image features generated by different segmentation lengths does not affect the model’s learning of fault features, which is also an advantage of fault diagnosis methods based on deep learning.

In the phase of signal conversion, after the original signals of different fault types are segmented, the continuous wavelet transform (CWT) [27,28,29], the short-time Fourier transform (STFT) [30,31], and the time-domain 2D image conversion methods are used to convert the segmented signals, and three different two-dimensional images are obtained. In order to accelerate the convergence and learning of the model, we normalized the transformed data with min-max, and used the normalized two-dimensional image as the input of the channel fusion stage. Finally, in the phase of channel fusion, three single-channel two-dimensional images are used as three channels of RGB images to form a new three-channel color image. Each pixel in a color image contains three components: R, G, and B. These three components are the values of three two-dimensional images at the same pixel position. As the final color image is the superposition of the original three-dimensional images, the fault features of each segment vibration signal are expressed in different fields, and the details and semantics of the fault features may be different. Therefore, compared with the single-channel processing method, the multi-channel fusion method can make the model better understand the characteristics of the fault samples, especially the subtle differences.

Below, we present the detailed steps for generating a three-channel color image:

Step 1: The original vibration signals of different health conditions are divided into equal intervals to form a series of segmented vibration signals, and the sample width is determined by the data points contained in the sample time interval;
Step 2: The segmented vibration signals are converted into a single-channel 2D time-frequency image using the CWT;
Step 3: The segmented vibration signals are transformed into a single-channel 2D time-frequency map using the STFT;
Step 4: The segmented vibration signals are transformed into a single-channel 2D time-domain image using the time-domain 2D image conversion method in Section 2;
Step 5: The 2D vibration images obtained by the three data processing methods noted above are fused by the channel fusion method. Then, the fused three-channel vibration image is taken as the input graph of the network. In the fused three-channel vibration image, each of the R, G, and B values of a pixel at entry (j,k), where 0 ≤ j < n, 0 ≤ k < m, equals the pixel value at entry (j,k) of each of the three single-channel 2D time-domain images.

### 3.2. Proposed SSJL Framework

**Figure 3:**
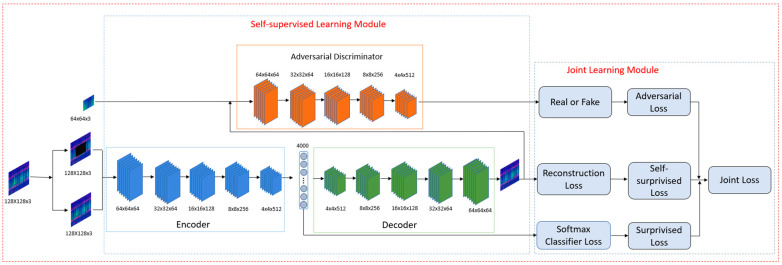
The architecture of the SSJL.

Figure 3 presents the SSJL framework presented in this paper, which includes a self-supervised learning module and a joint learning module. Compared with previous self-supervised learning algorithms, this paper proposes an end-to-end self-supervised learning method, which directly adds self-supervised learning to the fault diagnosis task. To express the potential fault features of unlabeled samples, two-stage self-supervised learning is avoided.

Image reconstruction is used as a self-supervised assistant task in the proposed algorithm framework. First, the unlabeled data are matted to be the input of the self-supervised module, based on the pretext task of image reconstruction, to learn the character of the object itself. The self-supervised module is composed of a generator and discriminator, and the generator component consists of an encoder and decoder. The encoder takes the image of the missing region as an input and generates the latent feature representation of the image. The decoder decodes the features learned by the encoder and generates the missing image content.

In the data decoding stage, the decoder and the encoder are connected through the channel’s fully connected layer [32], and the decoder structure is symmetrical to the encoder. To ensure the output and input spectra of the decoder are the same size, five deconvolution layers are used to sample the image. To ensure that the output feature of each layer is twice as large as the input feature, all deconvolution uses the same parameters to obtain a reconstructed image with the same size as the decoder input.

The main goal of the joint learning module is to realize the joint learning of labeled and unlabeled data. In this module, the potential universal features of unlabeled data are learned through self-supervised auxiliary tasks, and the specific features of fault signals in data are further learned through supervised learning. As shown in Figure 3, the optimization goal of joint learning is composed of supervised learning and self-supervised learning. The supervised loss is the standard cross-entropy loss, and the unsupervised loss is the self-supervised reconstruction loss. As can be seen from Figure 3, joint learning is a multi-task learning method; thus, the joint learning algorithm can more effectively find the optimal solution compared to the decision boundary supervised learning algorithm in a scenario of limited labeled data.

### 3.3. Optimization Goals

The SSJL method mainly involves self-supervised and supervised optimization, where self-supervised optimization corresponds to unlabeled data and supervised optimization corresponds to labeled data. The optimization objectives of the proposed method are described in detail below.

Self-Supervised Loss:

The self-supervised loss is mainly caused by the auxiliary task of image reconstruction. The context encoder is trained by recovering the missing part of the image. The self-supervised loss is mainly composed of two parts: the reconstruction loss (L2) and the counter loss.

The reconstruction loss is responsible for describing the overall structure of the missing area and the consistency of the missing area with the background. We use the normalized mask L2 distance as our reconstruction loss function $L_{rec}\left(x^{uld}\right)$, which is expressed by the following formula:

(1) Lrec(xuld)=‖⨀M^(xiuld−F((1−)M^⨀xiuld))‖22

where ⨀ represents the element-wise product operation of the corresponding position of the matrix (the element-wise product operation), $x_{i}^{uld}$ represents the number of the unlabeled input, and $\hat{M}$ represents the binary mask of the missing image (a pixel value of 1 is discarded, and a 0 is input). As the L2 loss will cause the decoder to generate a rough outline of the predicted object, it is usually unable to capture any high-frequency details. Therefore, the problem can be alleviated by increasing the antagonistic losses. The mathematical expression for fighting loss is as follows, the objective of which is to determine whether the input image is a real sample:

(2) $$L_{adv}=\underset{D}{max}E_{x^{uld}\in \chi }[log\left(D\left(x_{i}^{uld}\right)\right)+log\left(1-D\left(F\left(\left(1-\hat{M})⨀x_{i}^{uld}\right)\right)\right)\right]$$

where *F* represents a generator composed of an auto-encoder, and D represents a discriminator. The purpose of combating loss is to encourage the entire output of the context encoder to appear more realistic.

The overall self-supervised loss function of the model is defined as:

(3) $$L_{self}=\lambda_{rec}L_{rec}+\lambda_{adv}L_{adv}$$

where $\lambda_{rec}$ and $\lambda_{adv}$ represent the proportion of reconstruction loss and confrontation loss, respectively.

Supervised Loss:

The supervised learning part, in which a standard cross-entropy term is applied to the labeled samples as the optimization objective, is used to learn the accuracy of the labeled samples’ fault classification, which is expressed as follows [33]:

(4) $$L_{ld}=\begin{array}{c} min\\ \theta \end{array}\frac{1}{N}\overset{N}{\underset{i=1}{\sum }}y_{i}^{ld}log(P_{i}(y_{i}^{ld}|x_{i}^{ld}))$$

where the input data with labels is represented by $x_{i}^{ld}$, the label corresponding to the input $x_{i}^{ld}$ is represented by $y_{i}^{ld}$, the prediction probability of training samples is represented by $P_{i}$, and N is the total number of training samples.

Joint Loss:

The ultimate joint optimization objective of the proposed method is the sum of the self-supervised optimization objective and the supervised optimization objective:

(5) $$L_{joint}=\lambda_{self}L_{self}+L_{ld}$$

where $\lambda_{self}$ is the weighting factor, which represents the weight of the self-supervised loss in the overall loss. After errors and experimentation, this value was set to 0.5.

### 3.4. Fault Diagnosis Method Based on SSJL

In this paper, a new fault diagnosis method, SSJL, is proposed to address the problems of an insufficient quantity of labeled samples and the low accuracy of limited labeled samples in the field of industrial fault diagnosis. This method combines the improved self-supervised learning with three-channel vibration images, and uses a large number of unlabeled samples and finite labeled samples to learn fault features and diagnose faults. Superior diagnosis results were obtained compared with previous semi-supervised and supervised fault diagnosis methods.

In the process of model training, the joint learning loss is used as the objective function of the model, and the SSJL method is optimized using the gradient descent method. For unlabeled data, the self-supervised loss is used to evaluate the reconstruction error, and the cross-entropy classification loss is used to measure the classification results of different fault types. With the addition of self-monitoring loss, the feature extraction of unlabeled data can be realized by SSJL, and the cross-entropy classification loss further improves the accuracy of fault diagnosis. This method combines unlabeled data with labeled data using an end-to-end learning method, thus allowing better fault diagnosis to be achieved with a small amount of labeled data. The detailed training procedures of the proposed method are indicated as in Algorithm 1.
**Algorithm 1.** Training procedures of the SSJL method.**SSJL Algorithm****Input:**$\;\mathrm{Labeled}\;\mathrm{batch}\;X^{ld}=\{(x_{b}^{ld},\;y_{b}^{ld})\;:\;b\in \left(1,\ldots ,B\right)\},$$\mathrm{unlabeled}\;\mathrm{batch}X^{uld}=$ $\left\{(x_{b}^{uld},\;y_{b}^{uld})\;:\;b\in \left(1,\ldots ,B\right)\right\}$$,\;\mathrm{self}-\mathrm{supervised}\;\mathrm{loss}\;\mathrm{weight}\;\lambda_{self}$, reconstruction loss $\mathrm{weight}\;\lambda_{rec}$$,\;\mathrm{confrontation}\;\mathrm{loss}\;\mathrm{weight}\;\lambda_{adv}\;$;
 Parameter of training epoch Epoch Number; Iteration times in one epoch Batch Number;**for** e = 1 **to** Epoch Number **do****for** q = 1 **to** Batch Number **do**
// Self-supervised loss for unlabeled dataLadv=maxDExuld∈χ[log(D(xbuld))+log(1−D(F((1−)M^⨀xbuld)))]Lrec(xuld)=‖⨀M^(xbuld−F((1−)M^⨀xbuld))‖22$\;\;L_{self}=\lambda_{rec}L_{rec}+\lambda_{adv}L_{adv}$// Cross-entropy loss for labeled data$L_{ld}=\begin{array}{c} min\\ \theta \end{array}\frac{1}{B}\overset{B}{\underset{b=1}{\sum }}y_{b}^{ld}log(P_{b}(y_{b}^{ld}|x_{b}^{ld}))$// Joint loss$\;\;\;\;\;\;\;\;\;\;\;\;\;\;L_{joint}=\lambda_{self}L_{self}+L_{ld}$**end for****end for**

**Figure 4:**
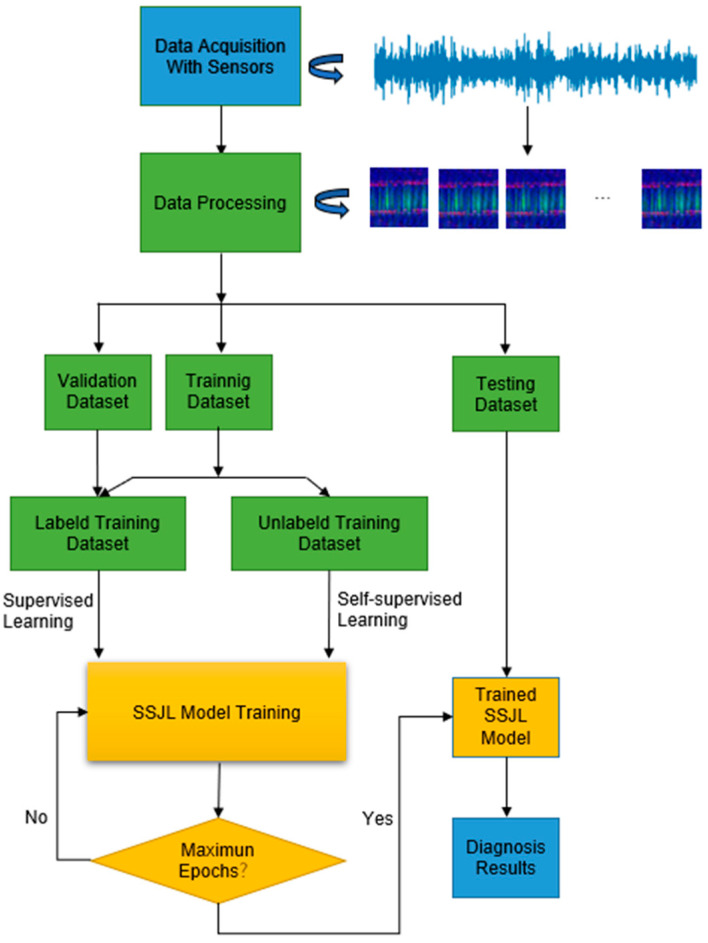
The flowchart of the SSJL method.

Figure 4 shows a fault diagnosis flow chart based on SSJL, in which vibration signals are measured by sensors mounted on an induction motor. The original vibration signal is then transformed into a three-channel vibration image as an input of SSJL using the data processing method presented in this paper, and the model is optimized by combining the supervised loss with the self-supervised loss until the algorithm converges. The detailed procedure for fault diagnosis is as follows:

(1) The use of the sensors to obtain the fault vibration signals from the bearing;
(2) The collected 1D time-domain signal is transformed into a three-channel vibration image using the data processing method presented in Section 2;
(3) The vibration image is divided into three parts according to a certain proportion: a training set, validation set, and test set. These are used in the training, verification, and testing of the model;
(4) A random sample is taken from the training set for marking; that is, the training set consists of several labeled samples and a large number of unlabeled samples;
(5) The training set is used to iteratively train the model until the model converges or reaches the maximum number of iterations;
(6) The test image set is input into the trained model to realize bearing fault diagnosis;
(7) The diagnosis result is output.

## 4. Experimental Results and Discussion

### 4.1. CWRU Dataset

#### 4.1.1. Description of CWRU Dataset

This section uses the motor bearing signals provided by Case Western Reserve University (CWRU) [34] to verify the effectiveness of the proposed method. The CWRU dataset consists of multi-variable vibration signals generated by the bearing test bench. The sampling frequency was 12 khz. The vibration data used in this study were obtained under four different health conditions: (1) normal state (N); (2) outer ring failure (OF); (3) inner ring failure (IF); and (4) ball failure (BF). Each fault type includes three types of single-point faults, each with a diameter of 0.07, 0.14, and 0.21 in., and each were processed using electric sparks. Therefore, the CWRU data set can be divided into 10 fault types without considering the different motor loads.

**Figure 5:**
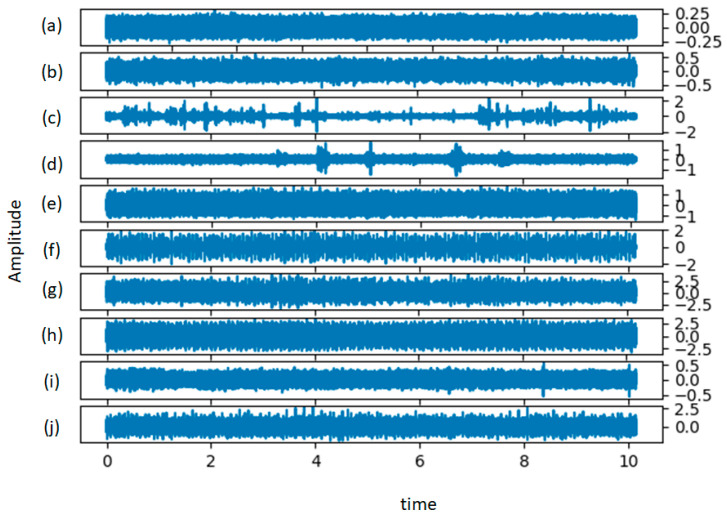
Raw data for (**a**) the normal for condition, (**b**) BF-007, (**c**) BF-014, (**d**) BF-021, (**e**) IF-007, (**f**) IF-014, (**g**) IF-021, (**h**) OF-007, (**i**) OF-014, and (**j**) OF-021.

Figure 5 shows the time-domain representation of the raw vibration data under each health condition. As the lengths of the signals acquired at the beginning were different, the signals were divided into small samples of the same length, as mentioned in Section 3.1. After splitting the signal processing, the sample was further converted into 128 × 128 × 3 vibration three-channel images. Finally, we obtained 35,473 samples, randomly selected from 21,994 training samples, 7449 validation samples, and 6030 test samples. The detailed description of the dataset is shown in Table 1.

#### 4.1.2. Model Parameters

In the process of model training, network parameters are initialized by Xavier. A backpropagation (BP) algorithm is used to update all parameters, and the Adam [35] optimization method is used. The input image size is set to 128 × 128 × 3 and the batch default is 64. The default values for the relevant parameters are shown in Table 2.

#### 4.1.3. Comparison of Different Data Processing Method

Three data processing methods were established to evaluate the effectiveness of the methods proposed in this study. In each of the comparison methods, only the data processing component was replaced, while other components were unchanged. The specific methods are detailed in Table 3. To simplify the expression, MC represents multi-channel, and SC represents single-channel.

Table 4 shows the diagnostic accuracy of the proposed method using different data processing methods. As shown in Table 4, CWT_SC has the worst diagnosis result, of only 89.82%. Compared with CWT_SC, the diagnostic accuracy of CWT_MC is improved by 2.32%; nonetheless, however, it is 7% lower than that of SSJL_MC. It is evident that SSJL_MC can be significantly improved compared with the previous method, which provides a foundation for the following model to achieve a higher accuracy of fault diagnosis.

**Figure 6:**
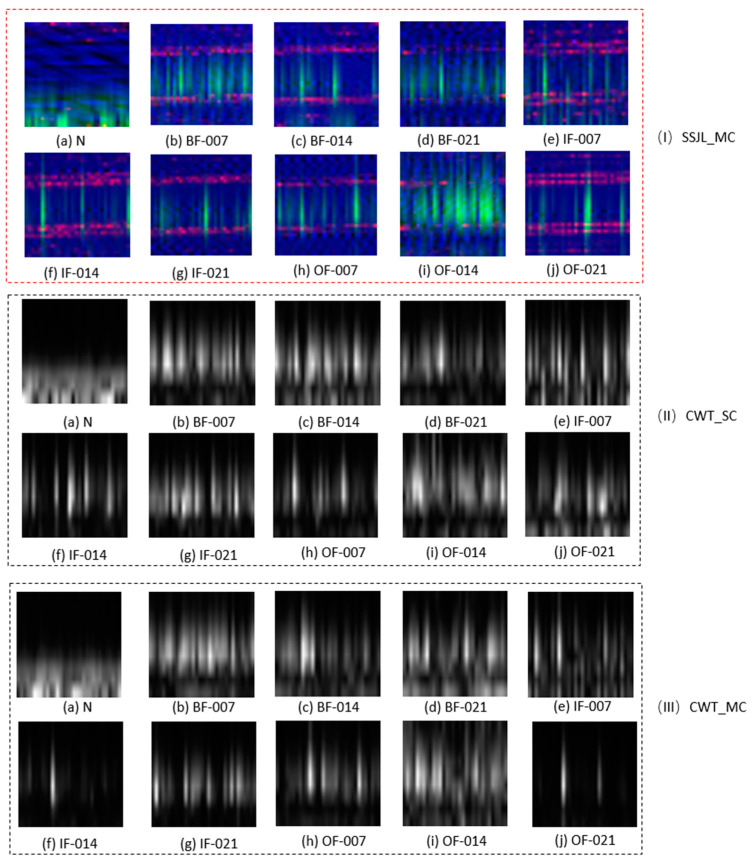
Vibration images obtained by different data processing methods: (**I**) SSJL_MC, (**II**) CWT_SC, and (**III**) CWT_MC.

Figure 6 shows the vibration images of each fault type diagnosis using different data processing methods. As shown in Figure 6, the image processing method SSJL_MC, is more effective than the other two methods at highlighting the difference between the fault types, as SSJL_MC uses different time-frequency analysis techniques to extract the time-frequency characteristics of the original signal. In addition, SSJL_MC combines the characteristics of the original time-domain signals, and the fault signals show different forms in different domains. Therefore, the SSJL_MC data processing method can obtain more comprehensive features of fault signals, prevent the omission of characteristic information, and achieve greater diagnostic accuracy. As shown in Figure 6III, the vibration images obtained using the CWT_MC method are not as distinguishable as those obtained by the SSJL method, mainly as each channel feature is only a copy of a single channel, and CWT_MC does not take into account the time-domain feature of the original signal. This may lead to the loss of useful fault characteristics. As a result, the CWT_MC-based fault diagnosis is not as effective as that of the SSJL_MC method. However, as shown in Figure 6II, CWT_SC showed the poorest result. It only uses a single-channel time-frequency transform to represent the fault features, which results in the lack of features of each fault type.

**Figure 7:**
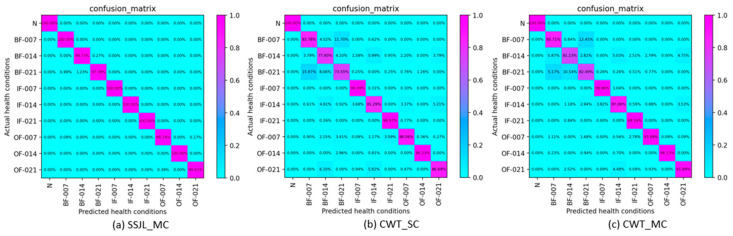
Confusion matrix of different methods: (**a**) SSJL_MC, (**b**) CWT_SC, and (**c**) CWT_MC.

To further verify the validity of the SSJL data processing method presented in this paper, Figure 7 shows the obfuscation matrix results of each data processing method on the test data set. As can be seen from Figure 7a–c, the identification rate of the SSJL_MC method was more than 99% for most fault types. In contrast, the recognition rates of the CWT_SC and CWT_MC methods were only greater than 99% for some fault types. This further indicates that the data processing method proposed in this paper ensures that the model learns more features of fault signals and, therefore, a higher performance of fault diagnosis is achieved.

#### 4.1.4. Influence of the Number of Labeled Samples in CWRU Datasets on Fault Diagnosis

To verify the validity of the proposed method, we evaluated the accuracy of fault diagnosis on a different number of labeled samples. For each working condition, the training samples were divided into labeled samples and unlabeled samples, in which the same number of samples were randomly selected as labeled samples, and the remaining training samples were unlabeled. In addition, we compared two classical algorithms in the field of fault diagnosis: one was the supervised learning method convolutional neural network, which omits the decoder used in the model presented in this paper; the other is a two-stage semi-supervised learning method DAE, which maintains the same model structure as that of the proposed method. As shown in Table 5, the fault diagnosis accuracy of the proposed method was evaluated in seven experiments with a different number of labeled samples. Table 5 shows the detailed results for diagnostic accuracy on the test dataset. It should be noted that, to reduce the impact of randomness in the training process, an average of 30 experimental results were used as the final result.

**Figure 8:**
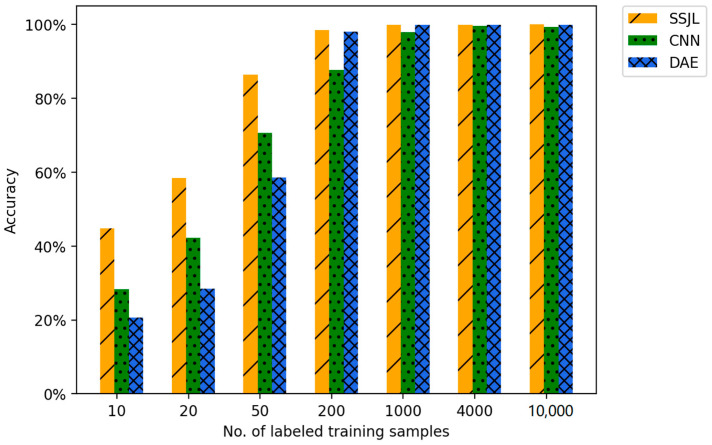
Accuracy comparison of different methods on the CWRU dataset.

As shown in Figure 8, the proposed SSJL achieves optimal fault diagnosis performance in all cases, and particularly in the case of few labeled data sets. As shown in Table 5, the diagnostic accuracy of SSJL for 10 health conditions of motor bearings was 58.48% when only two labeled training samples were used for each category. Under the same condition, the accuracy was 16.29% higher than that of CNN and 29.97% higher than that of DAE. The accuracy of the proposed method was 98.49% for 200 marker samples, whereas that of CNN was only 87.69%. This is mainly as CNN can only learn the fault features by optimizing the classification cross-entropy in the training process. This method can easily lead to over-fitting in small-scale training samples, and thus cannot obtain a good diagnosis effect. Table 5 shows that the fault diagnosis performance of DAE for 200 labeled samples is comparable to that of the proposed method, whereas the effect of DAE for a small number of labeled samples is inferior to that of the CNN. This is as the first stage of the two-stage DAE can only be learned from unlabeled samples, and the fine-tuning of labeled samples is only performed in the second stage when the number of labeled samples is low. Thus, this algorithm is more susceptible to the pre-training of unlabeled samples. As a result, the model cannot learn the detailed features of the fault samples, and is inferior to the CNN in the case of a small number of labeled samples. When the number of labeled samples reaches a certain number, the diagnostic effect of DAE based on the two-stage approach is significantly better than that of the CNN. As illustrated in Figure 8, the diagnostic accuracy of the three methods improves with the increase in the number of labeled samples. When the number of labeled samples was increased to 1000, the verification accuracy of all the methods was over 97%.

From the comparison results, it can be concluded that unlabeled samples help to improve the recognition performance. In addition, compared with other methods, the results show that the end-to-end joint learning method based on labeled and unlabeled samples is more effective in fault diagnosis tasks. In addition, the method proposed in this paper can not only achieve high diagnostic accuracy by using the self-supervised learning method, but also train the samples via the self-supervised task of image reconstruction. This enabled the model to search for common patterns and features in the training samples, thus reducing the risk of over-fitting the model.

#### 4.1.5. Influence of Unlabeled Sample Number in the CWRU Dataset on Fault Diagnosis

**Figure 9:**
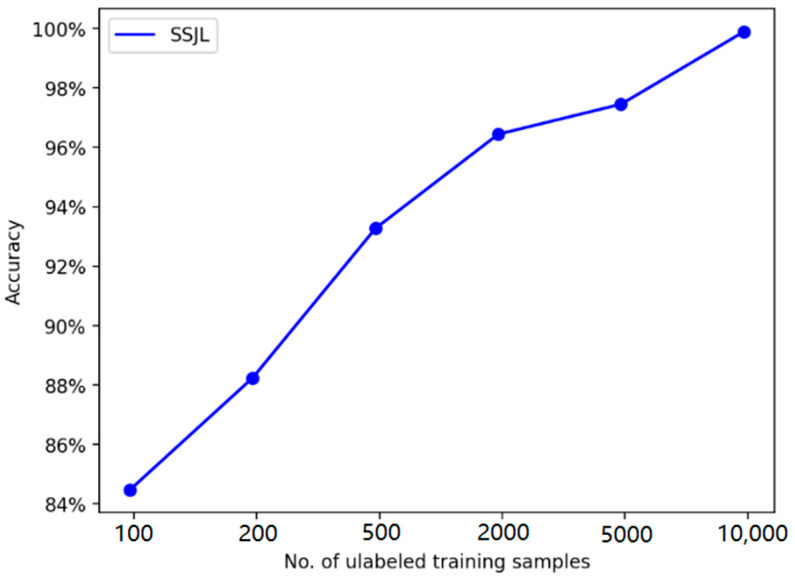
Diagnosis accuracies using a different number of unlabeled training samples on the CWRU dataset.

We further verified the effect of unlabeled sample size on the performance of the proposed method using different experiments. The number of labeled samples was 100, and the number of unlabeled samples ranged from 100 to 10,000. Table 6 shows the diagnostic accuracy of the proposed method on the test set. Figure 9 depicts the trend of test accuracy with the number of unlabeled samples. The final result was the average of 30 trials.

Figure 9 shows that, in the initial training stage, with the increase in unlabeled samples, the diagnostic accuracy of SSJL also increases. When the number of unlabeled samples reaches a certain number, the diagnostic accuracy rate no longer benefits from self-supervised learning, but stabilizes at the 99% level. Thus, the potential universal features of the fault samples can be learned from the unlabeled samples using self-supervised learning, which indirectly improves the accuracy of fault diagnosis, and the robustness and generalization ability of the model.

#### 4.1.6. Verification of the Effectiveness of the Proposed Method in the CWRU Dataset

**Figure 10:**
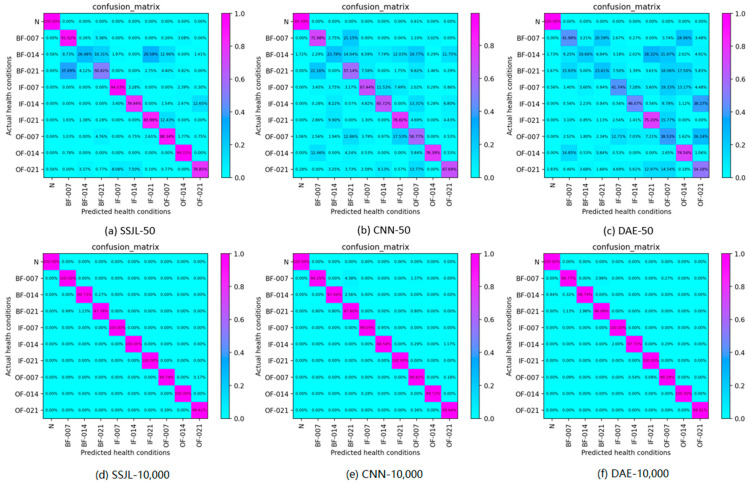
Confusion matrix for testing samples of the CWRU dataset: (**a**) SSJL-50, (**b**) CNN-50, (**c**) DAE-50, (**d**) SSJL-10,000, (**e**) CNN-10,000, and (**f**) DAE-10,000.

To further analyze the effects of a different number of labeled samples on the proposed method, Figure 10 shows the confusion matrix results of the methods when the number of labeled samples was 50 and 10,000. The confusion matrix represents the relationship between the true fault category and the predicted fault category, where the true fault category is represented by rows and the predicted fault category by columns. As can be seen from Figure 10a–c, when there were only five labeled samples in each category, the accuracy of the proposed method reached about 80% for most fault types, whereas the CNN only reached about 70%, and the accuracy of DAE was only about 60%. This fully demonstrates the advantages of SSJL in a small number of labeled samples, particularly in industrial scenarios in which labeled data are missing. As can be seen from Figure 10d–f, when the labeled samples reach a certain scale, all of the methods achieve an improved diagnosis result.

**Figure 11:**
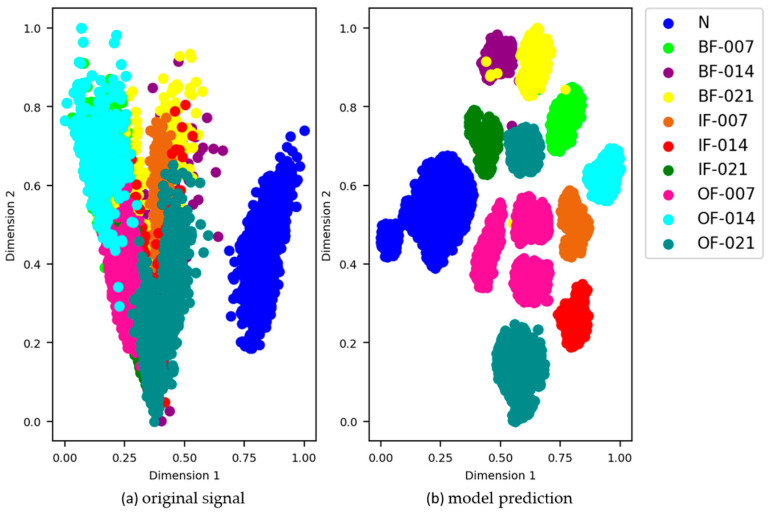
t-SNE representations of the CWRU testing samples: (**a**) original signal and (**b**) model prediction.

We used the t-SNE [36] method to visualize high-dimensional features in 2D space, and further verified the ability of automatic feature learning. Figure 11 shows the visual classification results for different fault types using the SSJL method when the number of labeled samples was 10,000, and Figure 11a,b represent the t-SNE representation of the original signal and model output characteristics, respectively. As shown in the figures, the 2D representation of the original signal has no distinct class-distinguishing boundary, and the fault classes are confused with one another. In contrast, after feature extraction by SSJL, the samples with the same health conditions are clustered together, the samples with different health conditions are separated, and the distribution of features is more organized. Experimental results show that this method can cluster the output features of test samples into 10 classes. This method can effectively improve the fault diagnosis ability by combining unlabeled and labeled samples.

### 4.2. MFPT Dataset

#### 4.2.1. Description of MFPT the Dataset

We used the MFPT rolling bearing dataset [37] provided by the Technical Institute of Mechanical Fault Prevention to verify the proposed method. The MFPT dataset consists of three sets of bearing vibration data: (1) a baseline set, in which each file is sampled at a rate of 97,656 SPS and lasts for 6 s; (2) an outer ring fault dataset, in which each file is sampled at a rate of 48,828 SPS and lasts for 3 s; and (3) an inner ring fault dataset, in which each file is sampled at a rate of 48,828 SPS and lasts for 3 s. All data were collected by a single-channel radial accelerometer.

**Figure 12:**
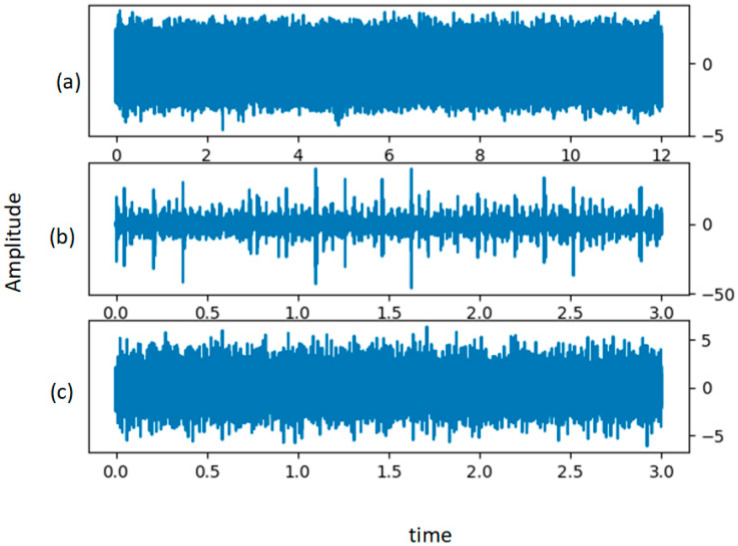
Raw data of groups (**a**) normal for condition, (**b**) outer, and (**c**) inner.

Figure 12 shows the time-domain diagram of the original vibration signals of MFPT under different health conditions. A total of 35,473 samples were obtained from the original MFPT dataset, of which 16,833 samples were randomly allocated for training, 3535 samples for verification, and 2681 samples for testing. A detailed description of the dataset is shown in Table 7.

#### 4.2.2. Influence of the Number of Labeled Samples in the MFPT Dataset on Fault Diagnosis

We also conducted a comparative experiment with a different number of labeled samples on the MFPT dataset, using the same model as that used in Section 3. Here, the minimum number of token samples was set to six and the maximum token data volume was set to 6000. As seen in Table 8, the fault diagnosis accuracy of SSJL was evaluated under different numbers of labeled samples. Table 8 shows the detailed results for diagnostic accuracy on the test dataset.

**Figure 13:**
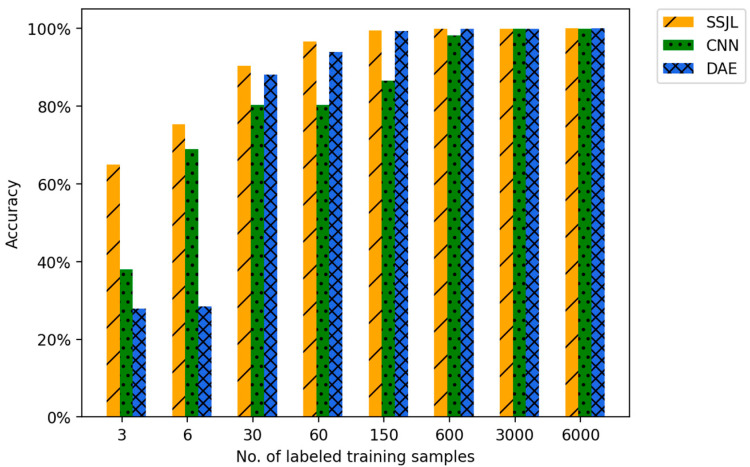
Accuracy comparison of different methods on MFPT dataset.

As shown in Figure 13, SSJL performed better than other comparable models in all cases. When there was only one labeled sample in each category, the accuracy of the proposed method was 64.91%, which was 26.85 percentage points higher than that of CNN and 37.02 percentage points higher than that of DAE. With the increase in the number of labeled samples, SSJL achieved an accuracy rate of 99.49% under 150 labeled samples, which was equivalent to the accuracy rate of the CNN algorithm with 3000 labeled samples. It can be concluded that the proposed algorithm can achieve or exceed the effect of supervised learning with fewer labeled samples, which solves the problem of marking fault data in an industrial environment. In addition, as in the case of the CWRU dataset, the DAE algorithm is not as accurate as the CNN in the case of a small number of labeled samples, and only exceeds the CNN when the number of labeled samples reaches a certain scale. In summary, the algorithm presented in this paper was the most advanced in all scenarios.

#### 4.2.3. The Effect of the Number of Unlabeled Data Points in the MFPT Dataset on Fault Diagnosis

**Figure 14:**
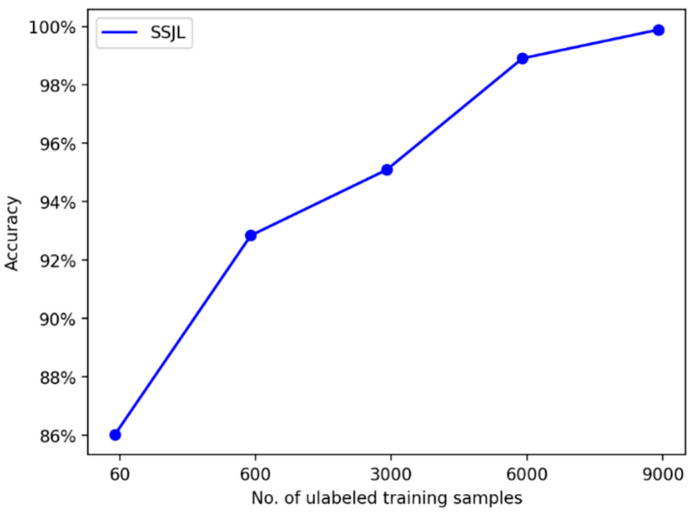
Diagnosis accuracies using different numbers of unlabeled samples on the MFPT dataset.

The result of the transformation on the verification accuracy due to the number of unlabeled training samples is shown in Figure 14. The number of labeled samples was set to 150, and the number of unlabeled samples ranged from 60 to 9000.

Similar to the results of the CWRU dataset, it can be seen that the diagnostic accuracy of the proposed method increases with the increase in unlabeled samples at the beginning of training. When unlabeled samples reach a certain number, the accuracy rate converges to a certain level.

#### 4.2.4. Verification of the Effectiveness of the Proposed Method in the MFPT Dataset

**Figure 15:**
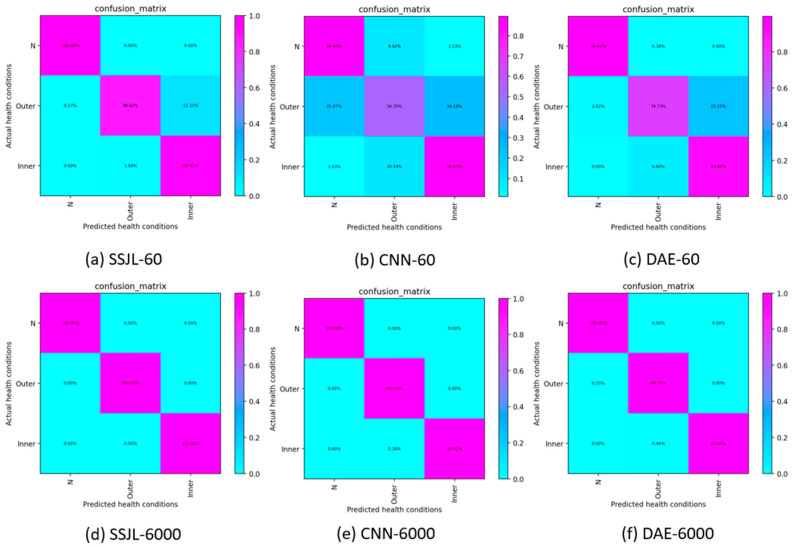
Confusion matrix on testing samples of the MFPT dataset: (**a**) SSJL-60, (**b**) CNN-60, (**c**) DAE-60, (**d**) SSJL-6000, (**e**) CNN-6000, and (**f**) DAE-6000.

The effectiveness of the proposed method was also verified on the MFPT dataset. As shown in Figure 15a–c, when the number of labeled samples was 60, this method was superior to other methods. In addition, Figure 15d–f shows that, as in the case of the CWRU dataset, all methods work well with an increasing number of labeled samples. This further verifies that the proposed method is competitive when used with different data sets.

**Figure 16:**
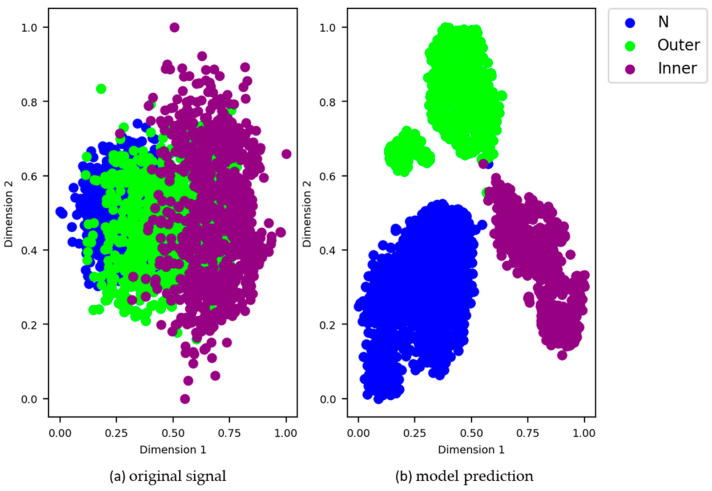
t-SNE representations of the MFPT testing samples: (**a**) original signal and (**b**) model prediction.

We also used the t-SNE [36] method to visualize high-dimensional features in two-dimensional space to verify model performance. Figure 16 shows the results of visual classification of different fault types on the test data set using the SSJL method when the number of marked samples was 6000. The scatter plots are colored differently depending on their true category. Figure 16a shows that all of the class characteristics of the original test data were mixed and there was no obvious boundary between the different classes. As shown in Figure 16b, the proposed depth model can be trained to aggregate the features of different classes and to distinguish the boundaries of different classes. This shows that our depth model retains a good feature extraction ability when trained using fewer labeled data sets.

## 5. Conclusions and Future Work

This paper presents a fault diagnosis method based on the combination of self-supervised and supervised learning analysis. In addition, the proposed three-channel vibration image preprocessing method can better highlight the local features of fault signals. The proposed method uses self-supervised learning to extract potentially universal features from unlabeled vibration samples and performs a self-supervised MSE loss operation on the label distribution of the original unlabeled samples. Furthermore, the supervised cross-entropy loss operation is used to improve the recognition performance. Two typical data sets of motor bearings were used to verify the analysis. The experimental results showed that our method is superior to common fault diagnosis methods, and that the advantage of the algorithm is more obvious when the number of labeled samples is small. When only five marker samples were used for each fault type in the CWRU dataset, the accuracy of this method reached 86.42%, and when 200 marker samples were used, the accuracy of this method reached 98.49%. Using 60 labeled samples on the MFPT dataset, the diagnostic accuracy was 96.61%. The results of this research show that our method is superior to common fault diagnosis methods for different numbers of labeled samples; in particular, when the number of labeled samples is small, the advantage of this method is more obvious. We also found that the number of labeled samples affects the accuracy of fault diagnosis; thus, an appropriate number of labeled samples can be chosen according to the actual situation. The experimental results showed that the proposed method can effectively address the over-fitting problem, and is more conducive to scenarios in which field data are missing. Furthermore, the comparison of different fault diagnosis data processing methods proved that the chosen method can enable the model to learn more features of fault signals and obtain superior fault diagnosis performance. The visualization results further verified the ability of the presented method to automatically learn features. The method can effectively address the problem of data annotation in practical industrial applications, and the end-to-end fault diagnosis method significantly reduces the complexity of model implementation. Thus, we believe that this approach is a promising fault diagnosis method that can be further extended to other mechanical fault diagnosis scenarios. In the future, we will further study the influence of different self-supervised learning mechanisms on fault diagnosis; that is, a fault diagnosis approach based on self-supervised learning will be the subject of our future work. In addition, the focus of the current paper is vibration image data after transformation. For this processing method, the correlation between the original vibration signals has not been fully considered. Thus, the direct application of self-monitoring to the original one-dimensional vibration signal will also be considered in future research. This will further promote the application of intelligent fault diagnosis in industrial scenarios.

## Figures and Tables

**Table 1:** Description of the CWRU dataset.

Health Condition	N	BF-007	BF-014	BF-021	IF-007	IF-014	IF-021	OF-007	OF-014	OF-021	35,473
Fault dimension(mm)	0	0.18	0.36	0.53	0.18	0.36	0.53	0.18	0.36	0.53	
No. of training samples	4630	1306	1346	1327	1352	1338	1329	3983	1319	4064	21,994
No. of validation samples	1597	466	457	445	446	460	446	1361	456	1315	7449
No. of testing samples	1296	374	347	377	353	349	373	1111	374	1076	6030

**Table 2:** The parameters used in this paper.

Parameter	Value
Epochs	150
Batch Size	64
Network Initialization	Xavier
Learning Rate	2 × 10^−4^
Optimization	Adam
Image Size	128 × 128 × 3

**Table 3:** Description of different data processing methods.

Methods	Description of the Data Processing Methods
SSJL_MC	Represents the data processing methods presented in this paper.
CWT_SC	Using traditional wavelet packet time-frequency transform, the input signal is converted into a 2D single-channel time-frequency map.
CWT_MC	Using the traditional wavelet packet time-frequency transform, the input signal is converted into a 2D multi-channel time-frequency graph (each channel is a copy of the single-channel time-frequency graph).

**Table 4:** Diagnostic results obtained by different data processing methods.

Methods	Average Accuracy	Standard Deviation
SSJL_MC	0.9999	4.1010
CWT_SC	0.8982	5.1104
CWT_MC	0.9214	5.0176

**Table 5:** Fault diagnosis accuracy of different methods on the CWRU dataset.

Method	No. of Labeled Training Samples						
	10	20	50	200	1000	4000	10,000
SSJL	0.4481	0.5848	0.8642	0.9849	0.9989	0.9997	1.000
CNN	0.2839	0.4219	0.7073	0.8769	0.9799	0.9968	0.9935
DAE	0.2065	0.2851	0.5862	0.9811	0.9987	0.9997	0.9999

**Table 6:** Diagnosis accuracies of different numbers of unlabeled training samples on the CWRU dataset.

Method	No. of Unlabeled Training Samples					
	100	200	500	2000	5000	10,000
SSJL	0.8447	0.8824	0.9327	0.9644	0.9745	0.9989

**Table 7:** Description of the MFPT dataset.

Class Label	0	1	2	Total
Health condition	N	Outer	Inner	
No. of training samples	4856	2777	2804	10,437
No. of validation samples	1624	991	920	3535
No. of testing samples	1310	753	798	2861

**Table 8:** Fault diagnosis accuracy of different methods on the MFPT dataset.

Method	No. of Labeled Training Samples							
	3	6	30	60	150	600	3000	6000
SSJL	0.6491	0.7536	0.9039	0.9661	0.9949	0.9997	0.9999	1.0
CNN	0.3806	0.6896	0.8036	0.8029	0.8661	0.9815	0.9993	0.9999
DAE	0.2789	0.2852	0.8819	0.9389	0.9941	0.9997	0.9999	1.0

## Data Availability

The data presented in this study are available on request from the corresponding author.
